# Performance and body composition effects of a pre-workout supplement and post-workout protein intake in trained crossfit individuals

**DOI:** 10.1186/1550-2783-10-S1-P28

**Published:** 2013-12-06

**Authors:** Stacie Urbina, Sara Hayward, Jordan Outlaw, Josh Holt, Bailey Burks, Brooke Cox, Eliza Faillace, Brittany Stai, Matthew Stone, Rob Wildman, Shawn Wells, Kristen Dunsmore, Abbie Smith-Ryan, Lem Taylor, Cliffa Foster, Colin Wilborn

**Affiliations:** 1 University of Mary Hardin-Baylor, Human Performance Lab, 900 College Street, Belton, TX 76531, USA; 2 Dymatize Nutrition Sport Performance Institute, Dallas, TX 75234, USA; 3 University of North Carolina Chapel Hill, Chapel Hill, NC 27599, USA

## Background

Despite widespread use of nutrition supplement s by CrossFit participants, existing data regarding performance and safety are minimal. Furthermore, increasing restrictions and drug testing in CrossFit, warrant the need for product specific research. The purpose of this study was to test the effects of a pre-workout supplement and post-workout protein & carbohydrate shake on CrossFit-specific performance measures and body composition.

## Methods

In an open label randomized study, 11 males and 13 females (n=24, mean ± SD; 32.71 ± 7.39 yrs, 173.15 ± 11.54 cm, 76.83 ± 15.77kg, 22.00 ± 9.73% body fat) who were regular CrossFit participants (≥6 months), and not currently taking ergogenic supplements, completed the study. Subjects were tested at baseline (T1) and 6 weeks (T2). Body composition variables including lean muscle mass (LBM), fat mass (FM), and percent body fat (BF) were assessed using DEXA (Hologic Wi). Performance variables: cardiorespiratory fitness (VO_2_max), Wingate peak power (PP), and mean power (MP) were tested 24-48 hours after completing two Workouts of the Day (WOD) with 20 minutes rest in between (WOD1: 500m row, 40 wall balls, 30 push-ups, 20 box jumps, 10 thrusters for time; WOD2: 800m run buy in, followed by 15-minutes as many rounds as possible of 5 burpees, 10 Kettlebell swings, 15 air squats) at T1 and T2. Subjects were matched based on sex and number of days they participate in CrossFit workouts per week, and then randomly assigned to the supplement (SUP) or control (CTL) group. SUP consisted of 19g of a pre-workout drink (pomegranate fruit extract, beet root extract, tart cherry extract, AssuriTEA™ green tea, and InnovaTEA™ black tea extract) 30 minutes before each CrossFit workout and post-workout supplement consisting of protein (40 g for females and 80g for males) and carbohydrate (80g for females and 160g for males). CTL subjects did not consume anything one hour before or after each workout. Participants were required to complete at least three workouts per week at a CrossFit gym, consume their supplement as directed, and complete mood state (MS), rate of perceived exertion (RPE), and delayed onset muscle soreness (DOMS) questionnaires. Data was analyzed by a group (SUP vs. CTL) × time (T1 vs. T2) repeated measures ANOVA (*p* <0 .05).

## Results

All data is presented as mean change scores. There were no time × group interactions for LBM (SUP 1130.86 ± 606.25 g; CTL 407.99 ± 728.42 g; p=0.36), FM (SUP 500.34 ± 437.82 g; CTL 107.77 ± 310.69 g; p=0.34) or BF (SUP 0.30 ± 0.21 %; CTL 0.06 ± 0.53 %; p=0.62). However, there was a significant trend for LBM (p = 0.063). There was no significant change in performance for VO_2_max (SUP -0.43 ± 1.88 ml/kg/min; CTL -1.525 ± 1 ml/kg/min; p=0.13), MP (SUP 6.54 ± 3.06 W; CTL 5.92 ± 2.91 W; p=0.58) or PP (SUP -8.76 ± 25.44 W; CTL 26.09 ± 21.74 W; p=0.54). Though there was no significant group × time interaction for WOD2 (SUP 17.08 ± 7.25 reps; 9.07% increase; CTL 4.91 ± 14.07 reps; 2.46% increase; p=0.23), there was a significant main effect for time (p=.037). A significant group × time interaction for WOD1 was observed (p =0.05; SUP -58.33 ± 52.31 seconds; 10.43% decrease; CTL -3.66 ± 14.38 seconds; 0.73% decrease).

## Conclusion

The combination of pre- and post-workout supplementation had no significant effect on improving body composition measures in trained CrossFit athletes. However, there was a significant improvement in WOD performance which is a critical consideration for competitive CrossFit athletes. Although not significant, the SUP yielded a 2.04% increase in LBM, which may be of practical significance for these athletes. This is the first study to investigate the potential benefit of a practical pre and post-WOD supplementation on CrossFit performance measures. Additional research is needed to better understand the potential for nutrition supplementation to optimize performance.

